# Treatment of non-tuberculosis mycobacteria skin infections

**DOI:** 10.3389/fphar.2023.1242156

**Published:** 2023-09-05

**Authors:** Xin-Yu Wang, Qian-Nan Jia, Jun Li

**Affiliations:** Department of Dermatology and Venereology, Peking Union Medical College Hospital (Dongdan Campus), Beijing, China

**Keywords:** non-tuberculosis mycobacteria (NTMs), skin diseases, infectious, anti-bacterial, mycobacterium infections, nontuberculous, atypical mycobacteria

## Abstract

Non-tuberculosis mycobacteria (NTM) skin infections have become increasingly prevalent in recent years, presenting a unique challenge in clinical management. This review explored the complexities of NTM infections localized to the superficial tissues and provided valuable insights into the optimal therapeutic strategies. The antibiotic selection should base on NTM species and their susceptibility profiles. It is recommended to adopt a comprehensive approach that considers the unique characteristics of superficial tissues to improve treatment effectiveness and reduce the incidence of adverse reactions, infection recurrence, and treatment failure. Infection control measures, patient education, and close monitoring should complement the treatment strategies to achieve favorable outcomes in managing NTM skin infections. Further efforts are warranted to elucidate factors and mechanisms contributing to treatment resistance and relapse. Future research should focus on exploring novel treatment options, innovative drug development/delivery platforms, and precise methodologies for determining therapeutic duration. Longitudinal studies are also needed to assess the long-term safety profiles of the integrated approaches.

## 1 Introduction

Non-tuberculosis mycobacteria (NTM) (Sharma and Upadhyay, 2020; Pavlik et al., 2022), mycobacteria other than *Mycobacterium tuberculosis* and *Mycobacterium leprae*, have emerged as a significant source of infections (Kumar et al., 2021; Nogueira et al., 2021). Among the diverse manifestations of NTM infections, skin and soft tissue involvements are prevalent clinical presentations. Although not posing an immediate life-threatening risk, these infections can result in significant morbidity and adversely affect the quality of life for affected individuals. Notably, there has been a global increase in reported cases of superficial NTM infections (Mei et al., 2019; Philips et al., 2019; Gopalaswamy et al., 2020) with contributing factors including the expanding population of immunocompromised individuals (Blakney et al., 2022; Toth et al., 2022) who face heightened susceptibility during injury or cosmetic procedures (Ahmed et al., 2020; Wang C. J. et al., 2022; Ni et al., 2023). Furthermore, the rise in NTM infections can be attributed, in part, to the ongoing adaptation of NTM to the human host. NTM’s remarkable capacity to thrive within the diverse skin environment, while effectively evading immune responses, also plays a role in the escalating incidence of these infections (Luo et al., 2021).

In light of these observations, understanding and effectively managing NTM skin infections are crucial for public health and patient wellbeing. Regarding treatment choices for NTM infections, various factors come into play due to the unique nature of these infections. Generally, the choice of treatment for NTM infections depends on the specific species and susceptibility pattern of the isolated organisms, as well as the severity and extent of the infection (Pennington et al., 2021). Current treatment strategies often reference the guidelines (Daley et al., 2020) of the Infectious Disease Society of America (IDSA) and American Thoracic Society (ATS) on pulmonary NTM-infected diseases. However, it should be noted that the ATS/IDSA guidelines for pulmonary diseases may not be directly applied to all skin-involved cases due to the unique characteristics of cutaneous NTM infections. The invasive or disseminated NTM infections may require a greater variety of drugs and a more extended treatment duration (Liu et al., 2023). When it comes to superficial involved cases, consideration must be given to the potential variations in pathogen types resulting from diverse infection pathways. The unique physiological structures and functions of the skin (Gravitz, 2018), compared to other anatomical sites, may also influence drug absorption and distribution, warranting tailored treatment approaches. In addition, the influence of local microbiota (Grice and Segre, 2011) and differences in host immune responses (Nguyen and Soulika, 2019) should not be underestimated, as they significantly impact treatment outcomes. Surgical interventions, phototherapy (Yang et al., 2022), and heat application (Lee and Lee, 2017) should also be considered as viable alternative treatment options due to anatomical variations in the infected sites. Furthermore, the emergence of newly invented antimycobacterial agents, such as MmpL3 inhibitors and Efflux Pump inhibitors (Rindi, 2020; North et al., 2023), are believed to have potent against slow-growing mycobacteria (SGM) and rapid-growing mycobacteria (RGM), also highlights the need for timely revision of treatment approaches. Finally, although NTM and *M. tuberculosis* shares similar physiological characteristics, virulence factors, and genetic drug targets (Rifat et al., 2021; Mei et al., 2023), it is still not advisable to fully copy the treatment regimens of TB. Many drugs being developed for treating TB do not exhibit any antimicrobial activity against NTM (Saxena et al., 2021). In summary, those factors highlight the need for updating of targeted treatment approaches to enhance skin-involved patient outcomes.

The management of cutaneous infections caused by various NTM subtypes poses significant challenges, necessitating a careful balance between therapeutic benefits and potential risks. The optimization of diverse treatment approaches, as well as the mitigation of adverse effects and infection recurrence, remain critical objectives. Through this comprehensive review, our aim is to provide an in-depth analysis of the treatment strategies for NTM skin infections and shed light on the complexities involved in addressing these clinical aspects.

## 2 Main text

### 2.1 Slow-growing mycobacteria (SGM)

SGM (>7 days for mature colony formation in solid media) mainly includes *Mycobacterium* marinum (*M. marinum*), *M. kansasii*, *Mycobacterium avium complex* (MAC), and many others (Yan et al., 2023). Common risk factors for SGM skin infections include exposure to contaminated water sources, such as swimming in contaminated water bodies or handling fish tanks, and skin injuries like cuts or scrapes that serve as entry points for the bacteria. Clinical presentations of SGM infections typically involve the development of nodules or raised skin lesions at the site of entry, which gradually enlarge and may lead to non-healing wounds or abscess formation (Riccardi et al., 2022). Infected areas may become painful and swollen, and the infection can spread along lymphatic vessels in a linear fashion. The final goal of the targeted treatment of SGM infection is to facilitate rehabilitation, shorten the treatment course, and prevent the pathogen from further progressing to deeper tissues to avoid multiple distributions.

#### 2.1.1 *Mycobacterium* marinum

*M. marinum* is the predominant pathogen responsible for SGM, often leading to skin and soft tissue infections (Hashish et al., 2018). Early diagnosis and prompt treatment of *M. marinum* infections pose significant challenges, especially during the atypical stage, potentially complicating the subsequent course of medication (Trčko et al., 2021). Presently, there is a lack of standardized norms concerning the selection, dosages, and treatment duration of drugs, as well as the consideration of surgery as an adjunct to treatment options (Seidel et al., 2022). According to the recommendations of IDSA/ATS guidelines, treatment typically involves using a combination of two active drugs, such as ethambutol-macrolide combinations, and continuing therapy until 1–2 months after symptom resolution. However, it is essential to acknowledge that no randomized controlled trials have been conducted in this domain, and the available data are insufficient to establish statistically significant evidence on drug efficacy and tolerability. The scarcity of verified data necessitates further research to comprehensively evaluate the effectiveness and safety of different treatment regimens for *M. marinum* infections.

##### 2.1.1.1 Susceptibility test and drug resistance characteristics of *M. marinum*

The *in vitro* drug sensitivity test (Hendrikx et al., 2022; Seidel et al., 2022) shows that *M. marinum* is moderately sensitive to streptomycin and resistant to azithromycin, isoniazid, and pyrazinamide. The minimum inhibitory concentrations (MICs) of levofloxacin, ciprofloxacin, and quinolones about *M. marinum* are high while keeping lower for rifampin, moxifloxacin, ethambutol, clarithromycin, linezolid, and tetracyclines. The results (Koushk-Jalali et al., 2019; Yeo et al., 2019; Castillo et al., 2020; Strobel et al., 2022) show that rifampicin, clarithromycin, sulfonamides, doxycycline, minocycline, and ethambutol are more suitable choices. Hence, given *M. marinum*’s susceptibility to numerous antibiotics, empirical treatment can be initiated at first, especially when susceptibility tests are unavailable (Oh et al., 2018). However, a test is needed when the condition is not improved after adequate treatment, or the mycobacterial culture is still positive after several months.

##### 2.1.1.2 Alone or combined use of antibiotics?

Several studies (Rallis et al., 2012; Chung et al., 2018; Franco-Paredes et al., 2018) indicated that oral monotherapy (single antibiotics such as clarithromycin, trimethoprim, and ciprofloxacin) is effective in immunocompetent patients only with superficial cutaneous *M. marinum* infections in the early stage, which also recommends that a suitable course of treatment need to last up 3–6 months, or the focus is limited and then proceed for 1–2 months (Aubry et al., 2017). Combined use of various active antimycobacterial agents is recommended under the involvement of deeper tissues, disseminated extracutaneous infection, and immunosuppressive status of hosts (Holden et al., 2018). Combinations, such as clarithromycin combined with rifampicin, clarithromycin combined with ethambutol, ethambutol combined with rifampicin, or three, are preferred (Strobel et al., 2022). The duration of therapy depends on the infection’s severity and treatment effects and could be extended moderately according to the host-drug interactions.

##### 2.1.1.3 Other treatments?

Surgeries (incision and drainage) are needed when the *M. marinum* goes deeper or poor curative effect for a long time. This may involve drainage of abscesses, removal of infected tissue, or excision of nodules or lesions that are unresponsive to antimicrobial therapy. Keeping the affected area clean and dry, avoiding activities that may traumatize the skin, and using appropriate dressings or bandages to protect the affected skin from further irritation or contamination are necessary. It is also recommended that amputation (Hendrikx et al., 2022) might be considered in case of severe cutaneous infections caused by multidrug-resistant isolates. Other than that, hot compress therapy (Strobel et al., 2022) might be a choice due to its high-temperature intolerance. (The optimal temperature is 30 Degrees Celsius).

#### 2.1.2 *Mycobacterium* kansasii

Cutaneous infections caused by *M. kansasii* predominantly affect immunocompromised hosts, including individuals with conditions such as diabetes or those who have undergone renal transplantation (Zhang et al., 2017; Okuno et al., 2020). Often, these cutaneous infections are concomitant with pulmonary involvement. As a result, when managing *M. kansasii* infections in superficial tissues, it is reasonable to refer to the guidelines established for the treatment of pulmonary NTM infections. According to the official ATS/ERS/ESCMID/IDSA clinical practice guidelines, for patients with rifampin-susceptible *M. kansasii*, a treatment course lasting over 12 months, comprising rifampicin, ethambutol, and either isoniazid or a macrolide, is advised.

There are a few differences between the guidelines of ATS/IDSA and the consensus of the British Thoracic Society (BTS) (Haworth et al., 2017). Rifampin and ethambutol are the same, while the part combined with isoniazid or clarithromycin differs. Both regimes mentioned above need years of treatment duration. A recent study (Chapagain et al., 2020) indicated that the regime of BTS and a novel one (rifapentine + tedizolid + minocycline) show better efficacy on *M. kansasii* of pulmonary diseases. Another study (Moon et al., 2019) found that the macrolide-containing regimen is as effective as the isoniazid-containing regimen, which might reduce the cumulative side effects of long-term use of anti-tuberculosis drugs. Clofazimine only shows a modest to poor impact on *M. kansasii* in a clinic in an observational study (Srivastava and Gumbo, 2018) on the antimicrobial effect of clofazimine monotherapy in the intracellular-infection hollow fiber model of *M. kansasii*.

Although the result of *in vitro* susceptibility testing of *M. kansasii* correlates little with clinical outcomes generally, it is still something to be learned from this. According to the effects of susceptibility tests (Zhang et al., 2017; Bakuła et al., 2018; Wang et al., 2019), *M. kansasii* is susceptible to ethambutol, rifampin, ethambutol, clarithromycin, aminoglycosides, and fluoroquinolones, which might guide clinical work of complicated cases. *M. kansasii* is resistant to pyrazinamide, and its potential resistance mechanism is not necessarily related to gene mutation but to great genetic diversity globally (Guo et al., 2022).

#### 2.1.3 *Mycobacterium avium* complex (MAC)

MAC (Daley, 2017), such as *M. avium*, *M. Intracellulare*, *M. Chimaera*, *M. indicuspranii*, is a group of SGM commonly identified in the respiratory system of patients with severely immunocompromised statuses (To et al., 2020). Given the circumstances, MAC infections often exhibit a propensity to disseminate from the initial infected site to involve other organs and tissues. Thus, the management of MAC infections affecting the skin and soft tissues warrants a comprehensive approach akin to treating invasive or disseminated cases (Chen et al., 2020; Crilly et al., 2020). A 3 year cross-sectional study (Akrami et al., 2023) found that MAC was susceptible to amikacin, moxifloxacin, and clarithromycin, while resistant to linezolid, rifampin, isoniazid, and clofazimine. In a cases report (Fukushi et al., 2022) of pulmonary and disseminated MAC patients confirmed by tissue-direct polymerase chain reaction-based nucleic acid lateral flow immunoassay, clinicians treated two patients with clarithromycin (CAM, 800 mg/day), rifampicin (RIP, 600 mg/day), and ethambutol (EB, 700 mg/day) for a year. No adverse side effects or recurrence were founded during the treatment. Omadacycline was tested as a potential treatment option for pulmonary MAC in hollow fibre system model, possibly as an alternative treatment for a new MAC regimen. The results of susceptibility testing in a retrospective study (Mok et al., 2019) of 88 isolates showed that *M. chimaera* is susceptible to clarithromycin, amikacin, rifabutin, and streptomycin while resistant to moxifloxacin and linezolid with a high probability, which might influence the overall therapeutic strategy. Several studies (Maurer et al., 2019; Li et al., 2022; Schulthess et al., 2023) on susceptibility testing found that *M. chimaera* and other members of the MAC generally have similar susceptibility (clarithromycin, amikacin, and rifabutin). In summary, for MAC infections that are susceptible to macrolides, a regimen of at least three drugs, including a macrolide and ethambutol, is preferred over a monotherapy of just a macrolide or ethambutol.

#### 2.1.4 Other less common SGM

Less common organisms include *M. xenopi, M. malmoense, M. simiae, and M. szulgai*. Despite the close phylogenetic relationship among these organisms, they exhibit discrete epidemiological characteristics and pathogenic behaviors. Therefore, the management of these SGM demand a nuanced approach that meticulously considers the distinctive attributes of each individual species. According to the established consensus, the recommended treatment approach involves a combination of two to three types of antibiotics, administered for a duration of at least 12 months beyond the point of culture conversion (Yan et al., 2023). For example, in the case of *M. xenopi* infections, a daily regimen that consists of at least three drugs: rifampicin, ethambutol, and either a macrolide or a fluoroquinolone (e.g., moxifloxacin) is recommended according to the official ATS/ERS/ESCMID/IDSA clinical practice guidelines. Nevertheless, the consensus regarding treatment recommendations (such as azithromycin, clarithromycin, rifampicin, ethambutol, amikacin, and moxifloxacin) for less common NTM species is largely based on low-quality evidence derived from published scientific literature.

### 2.2 Rapid-growing bacteria (RGM)

Similar to SGM infections, direct inoculation of RGM can occur through various routines, including trauma, surgical procedures, injections, tattoos, and other operations that involve the disruption of the skin barrier. In such instances, RGM can potentially spread to deeper tissues and cause infections beyond the initial site of entry. Clinical presentations of RGM skin infections often involve the development of nodules, abscesses, or ulcers at the site of entry. These skin lesions can be painful, red, and may contain pus. In immunocompromised individuals or those with underlying medical conditions, RGM skin infections can be more severe.

Treatment of RGM infections includes various regimens with different response rates (Kasperbauer and De Groote, 2015). The selection of antibiotics is mainly based on the results of drug susceptibility tests. According to the results (Chang and Whipps, 2015; Dumic and Lutwick, 2021), RGM is more sensitive to tigecycline, tobramycin, clarithromycin, and amikacin. However, the susceptibility profile varies from species to species. Drug resistance (Forbes et al., 2018; Shrivastava et al., 2020) still poses a significant challenge to a successful outcome due to the presence of the erm41 gene, which could lead to inducible resistance to macrolide, prolong the therapy, and increase the incidence of drug-induced toxicity. Below are details of the most common RGM species.

#### 2.2.1 *Mycobacterium* fortuitum complex

*Mycobacterium* fortuitum complex consists of *M. peregrinum*, *M. porcinum*, *M. fortuitum* and many others. Combined antibiotics treatment is often required, and surgical therapy may be needed optionally (Philips et al., 2019). After reviewing several *in vitro* antimicrobial susceptibility research (Forbes et al., 2018; Yeo et al., 2019; Da et al., 2020; Kumar et al., 2021; Das et al., 2022), we found that *M. fortuitum* strains were susceptible to many antibiotics. The isolates are susceptible to amikacin (intermediate to highly sensitive), ciprofloxacin (highly susceptible), doxycycline (intermediate susceptible), clofazimine, trimethoprim-sulfamethoxazole (TMP-SMX) and linezolid, resistance to all the antituberculosis agents, while different to macrolides (decreased sensitivity due to inducible susceptibility) and imipenem. A study (Chew et al., 2021) of 86 isolates showed that *M. fortuitum* is resistant to clarithromycin and tobramycin but susceptible to tetracyclines and quinolones. Similarly, a retrospective case series (Wang J. et al., 2022) of 18 patients with cutaneous *M. fortuitum* complex infections found that five uncomplicated infection cases showed an excellent response to the treatments. One patient received monotherapy of doxycycline for 8 weeks with no recurrence; the other four patients were treated with combined antibiotics, clarithromycin-minocycline, clarithromycin-ciprofloxacin, clarithromycin-TMP-SMX, and ciprofloxacin-TMP/SMX. Treatment courses range from 10 weeks to 40 weeks. While only three complicated infection patients with a prolonged period of the same therapy showed satisfactory clinical consequences, which meant immunosuppressed hosts were at higher risk of having persistent SGM infection than the immunocompetent population. Moreover, the findings from an *in vitro* and *in vivo* experiments (Ahmad et al., 2022) have demonstrated that gepotidacin, a first-in-class triazaacenapthylene topoisomerase inhibitor, exhibits a promising and potentially novel mechanism of action, allowing it to evade prevailing resistance mechanisms. These results underscore the potential of gepotidacin as a valuable therapeutic candidate with the ability to overcome resistance challenges commonly encountered with existing antimicrobial agents.

#### 2.2.2 Mycobacterium chelonae

The results of susceptibility testing (Franco-Paredes et al., 2018; Uslu et al., 2019; Watanabe et al., 2022) indicated that *M. chelonae* is often susceptible to macrolides, cefoxitin, fluoroquinolones, and tobramycin. The monotherapy (clarithromycin) can be sufficient for localized or superficial infections but not enough for patients who develop potential resistance. At least two antibiotic agents (oral macrolide combined with cefoxitin, amikacin, or imipenem) and 4–6 months of systemic treatment are recommended for these complicated cases. A biologics side-effects induced case (Frizzell et al., 2020) showed that omadacycline monotherapy at a dose of 300 mg orally daily for 4 months was efficient against *M. chelonae* skin and skin structure infections without recurrence in a 1-year follow-up. Surgical debridement, incising, draining, and source control are recommended in treatment if there is extensive involvement of extra-pulmonary *M. chelonae* infection (Dumic and Lutwick, 2021). Like *M. marinum*, thermal therapy was efficacious due to its thermal sensitivity. In addition, routine treatment (antimicrobial and surgical therapies) added a single bacteriophage (Little et al., 2022) showed stable disease improvement with no evidence of bacterial resistance to the phage. Bacteriophage therapy involves using viruses to infect and target specific bacteria, leading to the destruction and elimination of the bacterial population. Given the current challenges posed by antimicrobial resistance, bacteriophage therapy has emerged as a promising and attractive therapeutic option.

#### 2.2.3 *Mycobacterium* abscessus group

The *M. abscessus* group (*M. abscessus*, *M. massiliense*, and *M. bolletii*) is the primary source of cutaneous involvement of RGM (Jeong et al., 2017; Franco-Paredes et al., 2018). M. abscessus has an irregular resistance pattern to numerous anti-NTM agents (Lee et al., 2015; To et al., 2020; Kumar et al., 2021). Compared to *M. massiliense*, some *M. abscessus* and *M. bolletii* isolates (not all) have inducible macrolide resistance due to the functional erm41 gene, which could lead to inadequate response to a macrolides-dominant therapeutic schedule. Hence, antimicrobial susceptibility testing on all clinically significant isolates is strongly recommended before starting the therapy. The susceptibility list should include at least amikacin, cefoxitin, imipenem, clarithromycin, linezolid, doxycycline, tigecycline, ciprofloxacin, and moxifloxacin. Per the official ATS/ERS/ESCMID/IDSA clinical practice guidelines, for *M. abscessus* infections, whether the strains possess inducible or mutational macrolide resistance or not, it is recommended to initiate with a macrolide-inclusive multidrug regimen, which should encompass at least three drugs proven effective *in vitro*. An observational study (Da et al., 2020) showed that all strains of the *M. abscessus* group were susceptible to amikacin, linezolid, clofazimine, and tigecycline and suggested a prolonged drug resistance testing of 14 days to determine the presence of inducible resistance to macrolides is necessary. Monotherapy (clarithromycin) has shown promising efficacy in uncomplicated non-pulmonary disease, probably because its hand and foot lesions may represent a self-limited characteristic (Lee et al., 2015). However, invasive or disseminated *M. abscessus* and *M. bolletii* infections are complicated to treat; a combination of medication and a more comprehensive treatment course are necessary (Comba et al., 2021). Surgical resection of the infected tissues following chemotherapy to lessen the extensive progress might be a possible curative treatment for complex cases.

In the context of treating *M. abscessus* infection, the preclinical and clinical data derived from a study (Singh et al., 2023) suggest that the inclusion of omadacycline at a dosage of 300 mg per day in combination regimens holds promise for potential evaluation in Phase III trials involving patients with pulmonary involvement of *M. abscessus*. Such investigations could potentially bear significant guiding implications for addressing skin-related issues as well. Moreover, bacteriophages have also been explored as a potential therapeutic option. A study (Gorzynski et al., 2023) revealed that the lytic efficiency of phages is influenced by environmental factors, particularly when dealing with biofilm and intracellular states of *M. abscessus*. This observation has important implications, as it aids in the identification of therapeutic phages capable of reducing bacterial fitness by hindering antibiotic efflux function and attenuating the intrinsic resistance mechanisms of *M. abscessus* through targeted therapeutic interventions. Thiostrepton, a promising novel therapeutic drug candidate, has demonstrated substantial inhibition of *M. abscessus* growth in various contexts, including wild-type strains, subspecies, clinical isolates, and drug-resistant mutants, as evidenced by *in vitro* experiments and macrophage models. Additionally, it exhibited a dose-dependent reduction in proinflammatory cytokine production, suggesting its potential as an anti-inflammatory agent in the context of *M. abscessus* infection (Kim et al., 2019).

#### 2.2.4 Other RGM

According to the *in vitro* antimicrobial drug susceptibility testing, A study (Cantillon et al., 2022) using an open drug discovery approach found that oxazolidinones such as linezolid and doxycycline have excellent tissue penetration properties and are actively potent against *M. chimaera*. Two case reports (Shimizu et al., 2012; Wang C. J. et al., 2022) recommend combined therapy with adequate debridement and sensitive antibiotic administration for soft tissues in patients infected with *M. smegmatis*.

## 3 Discussion

Since more and more extrapulmonary NTM-infected cases have been reported recently and no unified treatment proposal could be referred to, a safer, more effective, higher adherent, a broader spectrum of anti-NTM activities, and more cutaneous-specific treatment strategy is needed. Thus, we reviewed the treatment of NTM infections involving skin or soft tissues in recent years to give some suggestions on this topic.

NTM skin involvements exhibit distinctive therapeutic disparities compared to other NTM-infected manifestations, owing to the unique structural characteristics of the skin, variations in drug distribution patterns, diverse modes of infection, relatively confined lesion distribution, milder disease severity, and a greater array of treatment modalities available. The management of NTM infections frequently entails the administration of multiple antimicrobial agents over extended durations, requiring vigilant clinical and laboratory monitoring. Nevertheless, the dearth of well-structured controlled trials investigating first-line treatment regimens, including optimal drug selection, dosage, and duration, poses challenges in formulating evidence-based guidelines for effectively managing a wide array of NTM species and associated diseases. Consequently, regimen selection should generally be guided by drug susceptibility testing. This testing involves assessing the susceptibility of the NTM isolate to various antimicrobial drugs, allowing for informed decisions on the most appropriate therapeutic approach. According to the results of drug sensitivity tests, our recommended treatment choices were summarized in Table 1. The establishment of rigorous clinical trials will be instrumental in addressing these knowledge gaps and facilitating the development of more effective and targeted treatment strategies for NTM skin infections. Waiting for species identification and susceptibility before treatment is reasonable without any delay for most superficial cases. However, the correlation between clinical outcomes and *in vitro* susceptibility thresholds remains undefined for the majority of NTM species (van Ingen et al., 2012a; Timmins, 2020). Different NTM subspecies have other susceptibility profiles to antimicrobial agents. The susceptible antibiotics against SGM differ from that of RGM (Alffenaar et al., 2021; Sharma et al., 2021). Therefore, subspecies level identification (no higher than the species level) and sensitivity testing of NTM, especially RGM, is recommended. For example, the Clinical and Laboratory Standards Institute (CLSI) recommends (Schoutrop et al., 2018) clarithromycin and amikacin susceptibility testing only for MAC, clarithromycin, and rifampicin for *M. kansasii*, and clarithromycin for *M. abscessus* complex. In addition, susceptibility testing should be prolonged as long as 6 weeks for SGM and 2 weeks for macrolides (Dartois and Dick, 2022). Recent advancements in molecular diagnostic techniques have improved the accuracy and speed of identifying NTM species and their drug susceptibilities, allowing for more precise and targeted treatment. However, over time, the stability of some antimycobacterial drugs is gradually affected by the pathogen, leading to a variable minimum inhibitory concentration (MIC), which then affects the final interpretation of the DST result. This partly explains why drug susceptibility testing results do not necessarily translate to a positive clinical response. The local microenvironments (Dey et al., 2010; Dartois and Dick, 2022), which can decrease therapeutic concentrations of drugs at the anatomical sites, might be another reason.

**TABLE 1:** The summary of preferred options for treating NTM skin infection.

Species		Recommended choices^a^	Unrecommended choices	Supplementary choices
Slow-growing mycobacteria (SGM)	*M. marinum*	Ethambutol, Azithromycin, Isoniazid, Pyrazinamide, Rifampicin, Clarithromycin, Sulfonamides, Doxycycline, Minocycline	Azithromycin, Isoniazid, Pyrazinamide, Levofloxacin, Ciprofloxacin, Quinolones	Surgery^b^ Hot Compress Therapy
	*M. kansasii*	Rifampin, Ethambutol, Clarithromycin, Clarithromycin, Aminoglycosides, Fluoroquinolones, Moxifloxacin	Clofazimine, Pyrazinamide, Linezolid, Isoniazid	Surgery
	MAC	Clarithromycin, Ethambutol, Amikacin, Rifabutin, Streptomycin	Linezolid, Isoniazid. Clofazimine, Rifampicin Moxifloxacin Linezolid	Surgery
	Other SGM	Azithromycin, Ethambutol, Ethambutol, Rifabutin	Unavailable	Surgery
Rapid-growing mycobacteria (RGM)	*M. fortuitum* complex	Amikacin, Ciprofloxacin, Doxycycline, Clofazimine, Trimethoprim-Sulfamethoxazole, Linezolid, Tetracyclines, Quinolones, Gepotidacin, Minocycline	Clarithromycin, Tobramycin Macrolides, Imipenem	Surgery
	*M. chelonae*	Clarithromycin, Cefoxitin, Fluoroquinolones, Tobramycin, Omadacycline	Macrolide (Inducible Resistant)	Surgery Bacteriophage Therapy
	*M. abscessus* complex	Amikacin, Linezolid, Clofazimine, Tigecycline, Clarithromycin, Omadacycline, Thiostrepton	Macrolide (Inducible Resistant)	Surgery Bacteriophage Therapy
	Other RGM	Amikacin, Linezolid, Doxycycline, Moxifloxacin, Moxifloxacin, Ciprofloxacin	Unavailable	Surgery

^a^ Monotherapy or combined therapy depends on the specific situation (NTM, species, infection sites, and disease severities).

^b^ The surgery operations include excision, debridement, drainage, and amputation, etc.

In addition, there is no well-defined treatment endpoint for superficial NTM infections. In contrast to TB or NTM pulmonary diseases, where the treatment endpoint can be determined by sputum specimen culture conversion and imaging results, defining the endpoint of treatment for superficial NTM infections remains uncertain. Typically, a treatment duration of 2–4 months is recommended for skin and soft-tissue NTM infections, while NTM pulmonary diseases often require at least 12 months of therapy after sputum culture reversion. To improve treatment efficacy while minimizing the risk of drug resistance, long-term and multidrug therapy is often necessary for NTM infections. However, this approach may lead to challenges such as drug interactions, drug-related adverse reactions (AEs), and high medication costs, potentially compromising treatment efficacy and patient compliance. Another method for determining the endpoint of treatment involves obtaining post-treatment specimens for culture to assess treatment efficacy, but this invasive procedure carries a heightened risk of reinfection, particularly in individuals with compromised immune systems. Finding a consensus on a specific and effective treatment endpoint for superficial NTM infections is imperative and demands further research and clinical investigation to ensure optimal patient outcomes and successful management of these challenging infections (Haworth et al., 2017; Wi, 2019). Currently, the determination of the treatment endpoint for skin NTM infection primarily relies on the assessment of changes in the patient’s clinical manifestations. These assessments typically involve evaluating the complete or substantial disappearance of preexisting skin lesions, the absence of new skin lesions, and the persistence of unchanged skin lesions after a specific duration of treatment. However, it is important to note that this criterion is subjective and lacks well-defined objective measures. To establish a more standardized and evidence-based approach for defining the treatment endpoint of skin NTM infection, further research and clinical investigations are necessary.

The low susceptibility of NTM to a wide range of antibiotics is attributed to their several characteristics. 1. Intrinsic resistance mechanisms: the first barrier is the unique metabolic condition [hydrophobicity of cell wall, and thereby low permeability (van Ingen et al., 2012a)] and the absence of porin or ABC transporter superfamily of the cell wall, which weakens the uptake and biotransformation of drugs and decreases the affinity with the drug target. 2. Inducible resistance mechanisms (Alffenaar et al., 2021): the second barrier is the genomic mutations of NTM, which could confer high-level resistance. Resistant strains are due to mutations at nucleotides. For instance, the changes of the 23S rRNA (functional erm genes) in *M. abscessus* isolates and of the RNA polymerase binding protein A (RbpA) in *M. smegmatis* are linked to the resistance to the macrolides and rifampicin (Dey et al., 2010). Comparative genomics and population genetics studies can provide insights into the genetic variability, evolution, and adaptation of NTM species. 3. Adaptative resistance mechanisms: he adaptability of NTM is the third barrier. NTM has extraordinary abilities in generation-upgrade time, and metabolic capabilities, which means they can adapt to stress before the cells are killed. They can form biofilms on the skin, which are complex microbial communities encased in an extracellular matrix. For example, one of the persistence strategies of NTM is hidden in biofilms (Slany et al., 2016), which generally leads to ten times less susceptibility to antibiotics than their counterparts. Understanding the mechanisms and dynamics of NTM biofilm formation on the skin is an active area of research, aiming to develop strategies to disrupt biofilms for improved treatment outcomes, including the use of biofilm-targeting agents and biofilm-disrupting techniques (such as enzymes, peptides, nanoparticles, and ultrasound). In addition, some studies (Huh et al., 2019) believe that NTM can enter a nonreplicating state and exhibit phenotypic drug resistance. However, up to now, the survey of resistance mechanisms associated with NTM still needs to be completed. Except for macrolides, the resistance mechanisms of many drugs still need to be clarified. It is essential to understand the basis for resistance and, more importantly, how to revise treatment choices to prevent the development of resistance.

**FIGURE 1:**
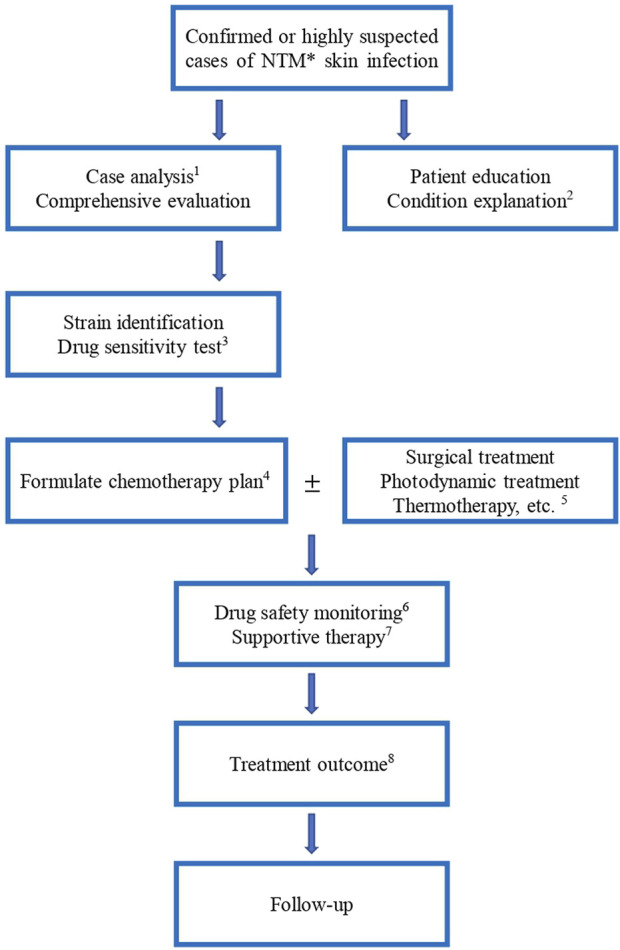
Procedure of NTM skin infection treatment. *: NTM, non-tuberculosis mycobacteria 1: The treatment should distinguish between mild and severe, drug resistance and non-resistance, initial and continuous stages, drug composition and dosage, children and adults, and HIV and non-HIV, etc. 3: Anti-NTM drugs include clarithromycin, azithromycin, ethambutol, amikacin, ciprofloxacin, moxifloxacin, rifampicin, rifampicin, isoniazid, cefoxitin, linezolid, chlorfazimine, tegacycline, imipenem/cilastatin, doxycycline, minocycline and compound sulfamethoxazole, etc. 4: Formulate the chemotherapy plan for NTM skin infections, the drugs should be selected according to the above-mentioned results. The type of medication and course of treatment are different for different NTM species. Experimental treatment of suspected NTM infections is not recommended. 5. Other treatment modalities are added depending on the patient’s condition. For patients with extensive lesions, abscess formation and poor drug efficacy, surgical debridement or foreign body removal can be actively used. 6. Monitor blood routine, liver and kidney function, blood electrolyte, urine routine, body mass, *mycobacterium* culture, hearing, visual field and color vision, electrocardiogram, etc. 7. Provide good patient education and explanation of the condition. For example, reduce contact with patients with NTM disease, and protect against human-to-human transmission. 8. Treatment outcome includes bacteriological negative conversion, bacteriological cure, clinical cure, cure, treatment failure, bacteriological recurrence, and death.

For uncomplicated skin-involved cases, primary empirical treatments and antibiotic monotherapy could respond well in most patients. Single-drug or combined (clarithromycin, rifampin, and ethambutol) treatments depend on the specific characteristics of the hosts, location, and identification of species. When a poor response to treatment or rapid progression is found, *in vitro* susceptibility testing should be addressed throughout the treatment. Compared to antimicrobial agents’ therapy alone, additional surgical operation of the localized infection with medication has proven to have better outcomes for extracutaneous involvement. Regular monitoring of the patient’s clinical response to treatment, as well as laboratory testing to assess the effectiveness of antimicrobial therapy, is important in the management of NTM skin infections. Follow-up appointments with the treating physician should be scheduled as recommended to monitor progress and make any necessary adjustments to the treatment plan. Our recommendations for treating NTM skin infections and recommended procedures are summarized in Table 2 and Figure 1.

**TABLE 2:** Recommendations on treatments of NTM skin infections.

	Recommendations
1. The characteristics required for novel anti-NTM drugs	1). Are ideally active against a broader spectrum of NTM; 2). Are bactericidal ideally against growing, and various drug-tolerant persist pathogens; 3). Could penetrate the multilayered structure of granulomas; 4). Drug interactions should be as minimal as possible
2. Choose the right treatment choices for each patient	After careful interpretation of the drug sensitivity results and the characteristics of the different cases, the choice of using a single therapy, a combination of antibiotics, physical therapy, or multiple parallel approaches is made
3. Drug monitoring and sensitivity tests are necessary	Therapeutic drug monitoring and prolonged drug sensitivity tests are always necessary during treatment. Clinical and laboratory monitoring of patients is essential. Treatment should be also tailored to the NTM species and susceptibility profile
4. Develop/screen drugs with the help of new platforms and new ideas	Referring to formal pharmacokinetics/pharmacodynamics research (such as CRISPR/Cas9 system and nanotechnology) might lead to safer and shorter-duration regimens. Novel molecular diagnostic technology can offer more effective, targeted multidrug treatments at the species level
5. Prevention and skin care are essential	Prevention is equally essential during percutaneous invasive operations or trauma. Immunosuppressed hosts need to pay more attention to infection during antimicrobial therapy. Proper wound care is also an important aspect of NTM skin disease management
6. Patient education and condition explanation	Counseling patients about the characteristics of NTM infections, such as choices of treatment, length of treatment, and possible side effects, to moderate their expectations for an unrealistic solution. Patients should also be advised to promptly report any new symptoms or changes in the affected skin to their healthcare provider
7. Multidisciplinary Cooperation	Multidisciplinary approach involving dermatologists, infectious disease specialists, and surgeons are recommended. Collaboration among healthcare professionals is important in determining the appropriate treatment plan, monitoring treatment response, and addressing potential complications

Emerging strategies are being explored to overcome drug resistance and improve treatment efficacy of complicated cases. 1. Screen existing drugs and new drugs: Novel antimicrobial drugs have shown promising activity against NTM species and may be considered in the treatment of NTM skin infections, particularly in cases where standard treatment regimens have failed or in the presence of drug-resistant strains. A study (Kaushik et al., 2019) showed that the new β-lactamase inhibitors relebactam and vaborbactam in combination with β-lactams have potent against *M. abscessus* complex clinical isolates *in vitro*. Clofazimine (Meir and Barkan, 2020), used for treating leprosy, is repurposed against *M. abscessus*. Besides, delamanid, pretomanid, and PIPD1 were also tested against *M. abscessus*. Telacebec is a promising novel drug with the potency of shorter duration and better tolerability (Lee and Pethe, 2022). However, the use of newer drugs may be limited by their potential side effects, higher costs, and availability. 2. Recombination of existing drugs: this is a very economical and efficient option. Previous studies (van Ingen et al., 2012b; Lee and Pethe, 2022) found that some antibiotics could increase cell wall permeability for the uptake of the second drug and accelerate durable cure, which indicates that the synergistic drug interactions could provide additional support in treating NTM infections. 3. Find new drugs according to new targets (Dartois and Dick, 2022): RNA polymerase, DNA gyrase, the ribosome, F-ATP synthase, and several enzymes. For instance, antibiotics that target oxidative phosphorylation energy-generate pathways could be a new choice. Alternatively, MmpL3 (Sethiya et al., 2020), a transporter crucial for exporting trehalose monomycolates to the periplasmic space and outer membrane, could also be a novel target in treating NTM. A study (Swain et al., 2021) found 15 new targets through screening 537 core proteins that researchers could further utilize to design inhibitors for discovering antimicrobial agents. In addition, some new drug research approaches are equally exciting. Macrophage infection assays, persister-specific assays, nonreplicating assays, biofilm assays, animal models, and lesion- or infection-site-specific pharmacokinetic assays, which can help us focus on skin and soft tissue, are instrumental methods to measure and evaluate effects when developing new anti-NTM drugs (Wu et al., 2018). For example, interferon-gamma, a cytokine that plays a role in the immune response against mycobacterial infections, has been used as an adjunct to antimicrobial therapy in some cases of NTM skin infections, particularly in patients with underlying immunosuppressive conditions. Other immune-enhancing agents, such as granulocyte-macrophage colony-stimulating factor (GM-CSF), have also been studied in the management of NTM infections. Fragment-based drug discovery (Togre et al., 2022) (FBDD) can concentrate on designing optimal inhibitors against potential therapeutic targets of NTM. A rabbit model could provide an acceptable surrogate model to study antibiotic penetration and simulate pharmacokinetic-pharmacodynamic tracks *in vivo* (Kaya et al., 2022).

Our article also has many limitations. First, randomized studies need to be added, and data regarding optimal treatment are limited. Clinical data on the efficacy of different treatment of NTM skin diseases in humans is limited, and further research is needed to determine its safety and effectiveness in clinical practice. Second, the resistance mechanism of NTM (genetic and pathogenic variations among species) infections needs to be understood more.

In summary, a comprehensive understanding of the various aspects discussed in this study is crucial for the effective management of cutaneous involvement caused by NTM. The ideal therapeutic approach should encompass a broader spectrum of anti-NTM activities while simultaneously considering the specific characteristics of cutaneous infections. The optimal treatment approach for NTM infections is still evolving, continuous research, clinical trials, and innovative therapeutic strategies are essential in the quest for safer, more effective, and tailored treatment options to combat NTM cutaneous involvement effectively. By addressing these aspects, clinicians can enhance patient outcomes and reduce the burden of NTM infections in affected populations.
